# Long-term survival of two patients with pancreatic cancer after resection of liver and lung oligometastases: a case report

**DOI:** 10.1186/s40792-020-01029-y

**Published:** 2020-12-07

**Authors:** Kei Hagiwara, Norifumi Harimoto, Kenichiro Araki, Akira Watanabe, Norio Kubo, Seshiru Nakazawa, Toshiki Yajima, Nobuyuki Uchida, Ken Shirabe

**Affiliations:** 1 Gunma University Graduate School of Medicine, Hepatobiliary and Pancreatic Surgery, 3-39-22 Showa-machi, Maebashi, Japan; 2 Division of General Thoracic Surgery, Gunma University Graduate School of Medicine, 3-39-22 Showa-machi, Maebashi, Japan; 3 Haramachi Red Cross Hospital, 698 Haramachi, Agatsuma, Gunma Japan; 4 Department of Hepatobiliary and Pancreatic Surgery, Gunma University Graduate School of medicine, 3-39-22 Showa-machi, Maebashi, Gunma 371-8511 Japan

**Keywords:** Pancreatic cancer, Oligometastases, Metastatic liver tumor, Metastatic lung tumor

## Abstract

**Background:**

The efficacy of resection of pancreatic cancer metastases has not been established. We here report two patients with long-term survival after resection of lung and liver metastases.

**Case presentation:**

The first patient underwent distal pancreatectomy for pancreatic cancer. One year later, she underwent partial hepatectomy for a single liver metastasis. She subsequently underwent pulmonary resections 7, 7.5, 9, and 10 years later for pulmonary metastases from pancreatic cancer. Thus, this patient underwent five surgeries for metastases, one for a liver metastasis and four for lung metastases. All of the tumors were pathologically diagnosed as metastatic pancreatic cancer. She is currently alive without new recurrence 10 years after the initial diagnosis. The second patient underwent pancreaticoduodenectomy for pancreatic cancer. Four years later, she underwent a thoracoscopic partial resection for lung metastasis. The tumor was similar to the pancreatic cancer on pathological examination. She is currently alive without new recurrences 6 years after the initial diagnosis.

**Conclusion:**

Long-term survival can be achieved in some patients with pancreatic cancer by resection of metachronous liver or lung metastases.

## Background

The term oligometastasis was introduced in 1995 and refers to a state of limited systemic metastases [1]. There is no precise definition of this term; however, most studies define oligometastases as the presence of up to three, or up to five, metastases [2]. Theoretically, local ablative therapy may be curative in some patients with oligometastasis [3].

Long-term survival after excision of metastases has been reported for some types of cancer. Resection of oligometastatic non-small-cell lung cancer can improve prognosis [4]. Resection of hepatic metastases from colorectal cancer has reportedly achieved good long-term cancer-specific survival benefit [5], repeat hepatic resection for colorectal cancer being as effective as primary surgical treatment [6].

However, pancreatic cancer is one of the most lethal cancers, being characterized by rapid progression, high metastatic potential, and limited response to conventional therapies [7]. Despite progress in the development of multidisciplinary treatments, including surgery, chemotherapy, and radiation therapy, the 5-year overall survival (OS) rate of patients with pancreatic cancer is less than 10% [8]. The efficacy of resection of metastatic pancreatic cancer has not been established; however, there is increasing interest in the possibility that metastasectomy in well-selected patients can prolong survival [9].

We here report two patients with pancreatic cancer with long-term survival after resection of liver and lung metastases.

## Case presentation

**Fig. 1:**
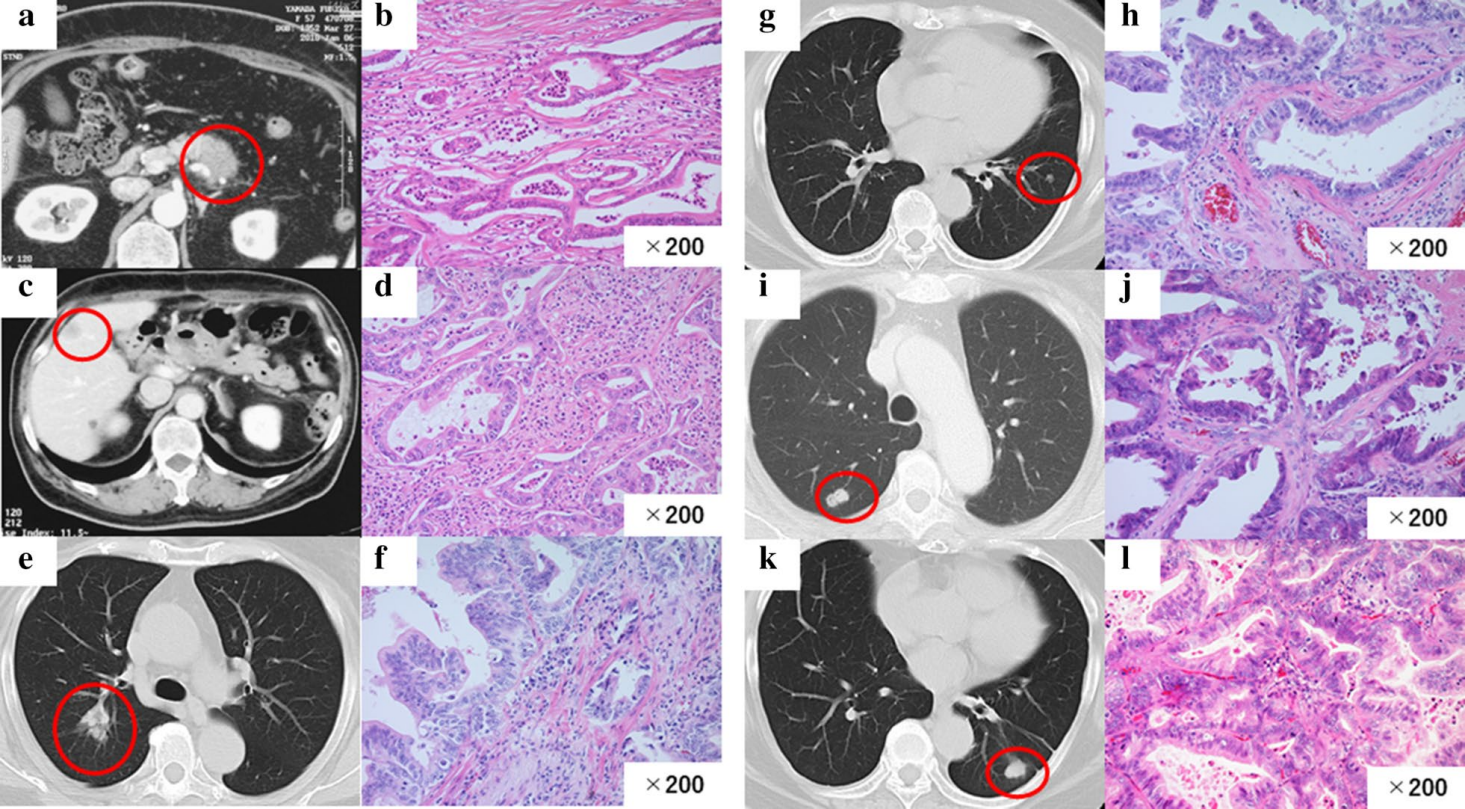
**a** CT image showing a 3.0 cm hypovascular tumor in the pancreatic body. **b** Photomicrograph showing cancer cells with abundant fibrosis are arranged as tubular structures (hematoxylin and eosin [HE] staining). Original magnification: ×200. **c** CT image showing a mass suggestive of metastasis in the liver (S5). **d** Photomicrograph showing cancer cells with abundant fibrosis that are similar to those of the original pancreatic cancer (HE staining). Original magnification: ×200. **e** CT image showing a nodule in the right lung (S2). **F.** Photomicrograph showing the tumor is similar to the original pancreatic cancer (HE staining). Original magnification: ×200. **g** CT image showing a nodule in the left lung (S9). **h** Photomicrograph showing the tumor is similar to the original pancreatic cancer (HE staining). Original magnification: ×200. **i.** CT image showing a nodule in the right lung (S6). **j** Photomicrograph showing a tumor that was diagnosed as metastatic pancreatic cancer (HE staining). Original magnification: ×200. **k** CT image showing a nodule in the left lung (S6). **l** Photomicrograph showing a tumor that was diagnosed as metastatic pancreatic cancer (HE staining). Original magnification: ×200

**Fig. 2:**
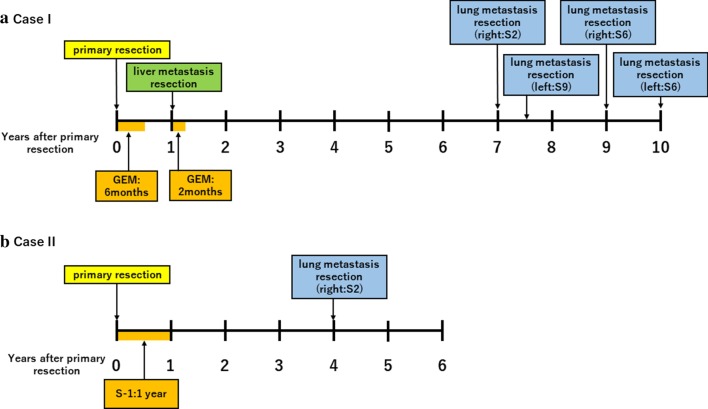
**a** Summary of the course of the case 1. **b** Summary of the course of the case 2

Case 1: A 57-year-old woman was admitted with cancer of the pancreatic body. Computed tomography (CT) showed a 3.0 cm hypovascular tumor in the pancreatic body and no evidence of intrahepatic, distant, or lymph node metastasis (Fig. 1a). This clinical course is summarized in Fig. 2a.

She underwent distal pancreatectomy with splenectomy, and lymph node dissection. On microscopic examination of the operative specimen, cancer cells with abundant fibrosis were arranged in tubular structures (Fig. 1b). There was microscopic evidence of invasion of the nerve plexus; however, no vascular invasion was identified and the resection margin was negative. The histopathological diagnosis was moderately differentiated tubular adenocarcinoma (pT3N0M0 Stage IIA: UICC).

One year after resection of the primary tumor, a mass suggestive of liver metastasis was identified (S5) (Fig. 1c). No distant metastases were detected. She underwent a partial hepatectomy for liver metastasis. On microscopic examination, the cancer had abundant fibrosis and was similar to the original pancreatic cancer (Fig. 1d). The tumor was diagnosed as metastatic pancreatic cancer.

She was followed up with no recurrence for the next 5 years. However, 7 years after the primary resection, CT showed a 1-cm nodule in the right lung (S2) (Fig. 1e) that was diagnosed as a lung metastasis from pancreatic cancer. The single tumor appeared to be resectable so thoracoscopic resection of the right upper lobe was performed. The tumor was similar to the original pancreatic cancer on microscopic examination and was diagnosed as metastatic pancreatic cancer (Fig. 1f).

Seven years and 6 months after the primary resection, she underwent thoracoscopic partial resection of a nodule in the left lung (S9) (Fig. 1g). This tumor was also diagnosed as metastatic pancreatic cancer (Fig. 1g). Nine years after the primary resection, she underwent partial resection of the right lobe (Fig. 1i) for another tumor that was diagnosed as metastatic pancreatic cancer (Fig. 1j). Ten years after the primary resection, she underwent thoracoscopic resection of the left lower lobe (Fig. 1k) for another tumor that was also diagnosed as metastatic pancreatic cancer (Fig. 1l). She has since been followed up with no evidence of recurrence and remains alive without new recurrence 10 years after the initial diagnosis. Thus, she underwent five surgeries, one for liver metastasis and four for lung metastases after the primary resection.

Changes in the tumor markers carcinoembryonic antigen (CEA) and carbohydrate antigen 19-9 (CA19-9) before and after surgical resections are summarized in Table 1. The concentrations of CA19-9 had increased slightly before hepatectomy for metastatic liver cancer; however, the concentrations of these tumor markers were within their normal ranges at the times of the remaining measurements.

**Table 1:** Summary of changes in tumor markers concentrations

	Preoperative		Postoperative	
	CEA (ng/mL) (0–5.0)	CA19-9 (U/mL) (0–37)	CEA (ng/mL) (0–5.0)	CA19-9 (U/mL) (0–37)
Case 1				
Primary resection	1.7	14	1.3	12
Liver metastasis resection	1.5	61	1.5	12
First lung metastasis resection	2.9	20	–	–
Second lung metastasis resection	2.7	–	–	–
Third lung metastasis resection	3.0	–	–	–
Fourth lung metastasis resection	3.0	18	–	–
Case 2				
Primary resection	2.2	104	2.6	24
Lung metastasis resection	5.7	47	5.5	53

Gemcitabine (GEM) had been administered intravenously for 6 months after the primary resection as adjuvant chemotherapy, this having been the standard regimen at that time. However, she had experienced severe adverse effects, namely nausea and general malaise. Although adjuvant chemotherapy using GEM for 6 months after resection of the liver metastasis had been planned, she only received this chemotherapy for 2 months because of its severe adverse effects. After every pulmonary resection, adjuvant chemotherapy with S-1 (tegafur/gimeracil/oteracil) for 6 months was proposed, but she consistently refused it.

**Fig. 3:**
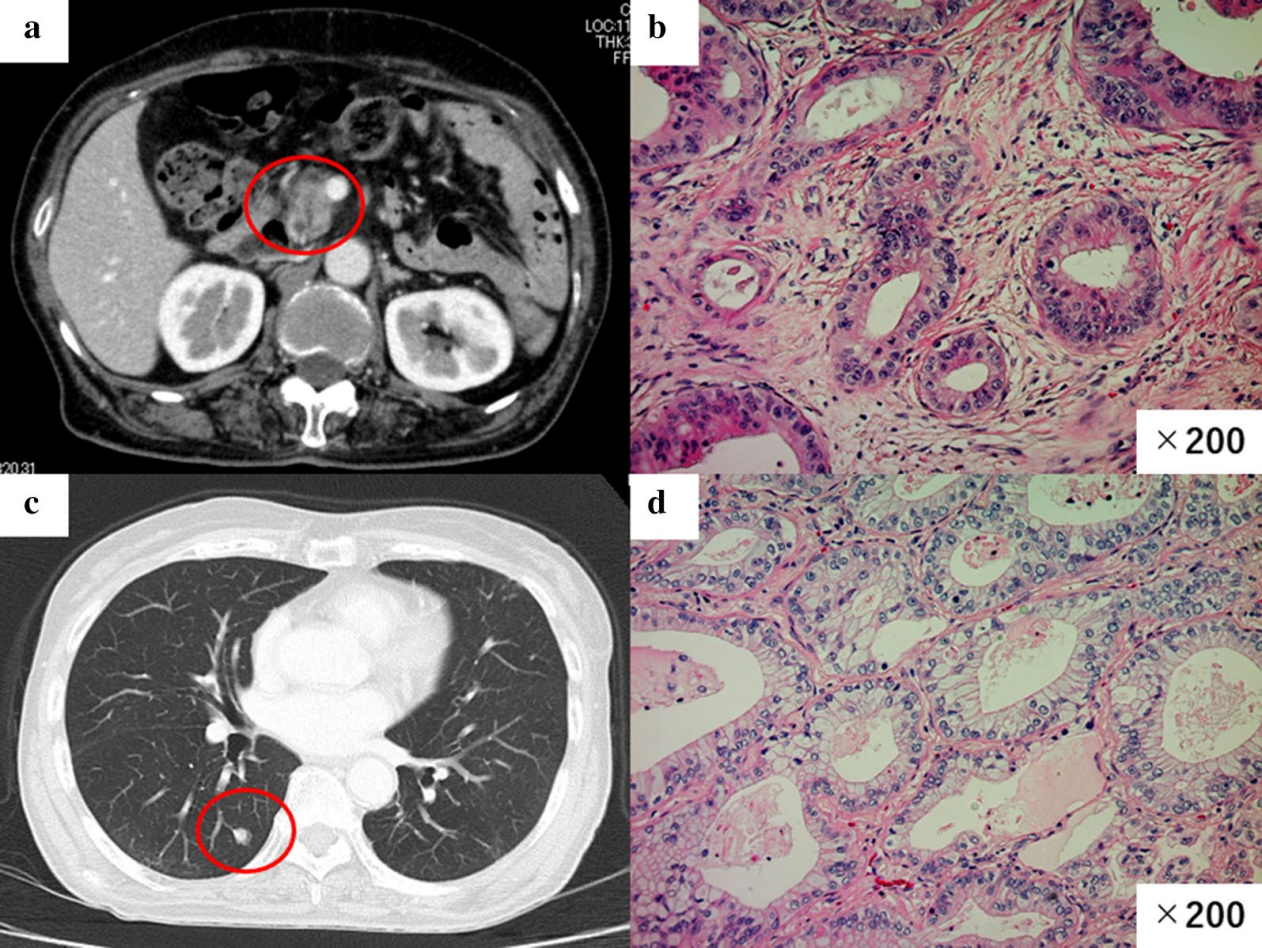
**a** CT image showing a 2.0 cm hypovascular tumor in the pancreatic head. **b** Photomicrograph showing cancer cells with abundant fibrosis arranged in tubular structures (HE staining). Original magnification: ×200. **c** CT image showing a nodule in the right lung (S6). **d** Photomicrograph showing a tumor that was diagnosed as metastatic pancreatic cancer (HE staining). Original magnification: ×200

Case 2: A 73-year-old woman was admitted with cancer of the pancreatic head. CT showed a 2.0 cm hypovascular tumor in the pancreatic head and no evidence of intrahepatic, distant, or lymph node metastasis (Fig. 3a). This clinical course is summarized in Fig. 2a.

She underwent pancreaticoduodenectomy and lymph node dissection. Microscopic examination of the operative specimen showed cancer cells with abundant fibrosis arranged in tubular structures (Fig. 3b). Cancer cells were invading the nerve plexus and lymph vessels; however, no vascular invasion was identified and the resection margin was negative. One lymph node metastasis was detected. The histopathological diagnosis was moderately differentiated tubular adenocarcinoma (pT3N1M0 Stage IIB: UICC).

Four years after the primary resection, CT showed a nodule in the right lung (S6) (Fig. 3c). We performed a thoracoscopic partial resection of the right lobe. The tumor was found to be similar to the pancreatic cancer on microscopic examination and was diagnosed as metastatic pancreatic cancer (Fig. 3d). She has been followed up with no recurrence and remains alive without new recurrence 6 years after the initial diagnosis.

Changes in the tumor markers CEA and CA19-9 before and after surgical resections are summarized in Table 1. The concentrations of CA19-9 had increased slightly before primary resection of the pancreas, and had fallen to within the normal range after resection. CA19-9 concentrations were slightly increased both before and after pulmonary resection.

She underwent adjuvant chemotherapy with S-1 for 1 year after the primary resection. Chemotherapy with S-1 for a further 6 months was proposed after resection of her lung metastasis; however, she refused this.

## Discussion

Pancreatic cancer, one of the most lethal cancers, is characterized by rapid progression and limited response to conventional therapies [7]. Despite progress in the development of multidisciplinary treatments, the 5-year OS rate of patients with pancreatic cancer is less than 10% [8]. Metastatic or recurrent cancer is difficult to manage; in particular, surgery alone appears to be non-curative in patients with recurrent pancreatic cancer [9]. The most effective treatment for pancreatic cancer is surgical resection; however, in patients with simultaneous metastases at the time of diagnosis, the disease is unresectable according to the National Comprehensive Cancer Network [10]. In addition, resection is rarely indicated for metastases from pancreatic cancer. Isabella et al. reported administering systemic chemotherapy to 535 patients with oligometastasis from pancreatic cancer, only 24 (4.5%) of whom underwent successful resection [11]. However, resection of metachronous oligometastasis in well-selected patients with pancreatic cancer is reportedly effective [12]. The prognosis of pancreatic cancer reportedly improves when metachronous liver and lung metastases are resected, median overall survival after the first treatment varying from 51 to 121 months for lung metastases and from 24 to 40 months for liver metastases [13]. Isabella et al. reported a median OS of patients with pancreatic cancer undergoing resection of oligometastases of 56 months (range 36–75) [14], whereas Wright et al. reported a median OS for such patients of 18.2 months (95% CI 11.8–35.5) [15]. Still, our patients who underwent surgical resection for metastases, who survived more than 120 months and 72 months after the initial diagnosis, are extremely rare.

The characteristics of patients with long-term survival after resection of oligometastasis have not been fully clarified. In a study in which patients underwent pulmonary resection for suspected metastasis after resection of a primary pancreatic cancer, those with late recurrence (later than 17 months after primary surgery) had better OS than those with earlier development of pulmonary nodules (32.2 vs. 14.75 months, p = 0.025). High serum concentrations of tumor markers (CA 19-9 and CEA) and number of pulmonary metastases reportedly have no significant impact on outcome [16]. Lu et al. reported that resection of solitary hepatic or pulmonary metastases from pancreatic cancer should be considered only when: (I) complete surgical resection (R0) can be achieved by pancreatectomy; (II) the pancreatic cancer has responded to neoadjuvant chemotherapy; (III) the oligometastases are resectable; and (IV) the patient is in overall good general health with few comorbidities [17]. Both of our patients met conditions (I), (III) and (IV); however, neither of them received neoadjuvant chemotherapy. The reasons for our failure to prescribe this treatment were as follows: (1) the concept of neoadjuvant chemotherapy had not been clarified at the time in question; (2) there were few recommended chemotherapeutic regimens at that time; and (3) both patients refused chemotherapy because they had experienced severe adverse effects while receiving previous adjuvant chemotherapy.

Nowadays, the prognosis of unresectable pancreatic cancer has greatly improved with the advent of effective chemotherapeutic regimens such as FOLFIRINOX and GEM/nab-paclitaxel [18]. These treatments have been reported to have tumor-reducing effects [19, 20]. A recent study suggested that neoadjuvant chemotherapy might be important in determining surgical indications for oligometastasis, especially when it has occurred soon after resection of the primary lesion [11, 16, 17]. Thus, if a single liver metastasis occurs within 1 year of primary resection, neoadjuvant chemotherapy using effective chemotherapeutic regimens is currently considered for that metastasis. Additionally, more oligometastases may become resectable as a result of administering these treatments. Nevertheless, some patients who undergo surgical resection of oligometastasis experience long subsequent survivals without chemotherapy. Further investigation is necessary to determine the utility of neo and/or adjuvant chemotherapy for oligometastasis.

Systematic follow-up of patients who undergo surgical resection of oligometastasis is very important. Although one study has reported that up to 80% of patients who have undergone resection of oligometastases of pancreatic cancer develop recurrence within 2 years [12], there is no standard recommendation as to when follow-up can be stopped. The present cases indicate that late recurrence can occur and life-long follow-up may be necessary.

When treating metastases from pancreatic cancer, it is necessary to consider the site, number of metastases, general condition of the patient and the necessity of chemotherapy to determine whether resection is indicated.

## Conclusions

We here present two patients with pancreatic cancer who achieved long-term survival by repeated resection of metastatic lesions. Resection of metachronous liver or lung metastases can result in long-term survival of some patients with pancreatic cancer.

## Data Availability

The data supporting the conclusions of this article are included within the article.

## Abbreviations

CA19-9: Carbohydrate antigen 19-9
CEA: Carcinoembryonic antigen
CT: Computed tomography
GEM: Gemcitabine
HE: Hematoxylin and eosin
OS: Overall survival
UICC: S-1: tegafur/gimeracil/oteracil; Union for International Cancer Control
